# Money Does Not Always Buy Happiness, but Are Richer People Less Happy in Their Daily Lives? It Depends on How You Analyze Income

**DOI:** 10.3389/fpsyg.2022.883137

**Published:** 2022-05-31

**Authors:** Laura Kudrna, Kostadin Kushlev

**Affiliations:** ^1^Institute of Applied Health Research, University of Birmingham, Birmingham, United Kingdom; ^2^Department of Psychology, Georgetown University, Washington, DC, United States

**Keywords:** happiness, measurement, time use, income, methodology

## Abstract

Do people who have more money feel happier during their daily activities? Some prior research has found no relationship between income and daily happiness when treating income as a continuous variable in OLS regressions, although results differ between studies. We re-analyzed existing data from the United States and Germany, treating household income as a categorical variable and using lowess and spline regressions to explore nonlinearities. Our analyses reveal that these methodological decisions change the results and conclusions about the relationship between income and happiness. In American and German diary data from 2010 to 2015, results for the continuous treatment of income showed a null relationship with happiness, whereas the categorization of income showed that some of those with higher incomes reported feeling less happy than some of those with lower incomes. Lowess and spline regressions suggested null results overall, and there was no evidence of a relationship between income and happiness in Experience Sampling Methodology (ESM) data. Not all analytic approaches generate the same results, which may contribute to explaining discrepant results in existing studies about the correlates of happiness. Future research should be explicit about their approaches to measuring and analyzing income when studying its relationship with subjective well-being, ideally testing different approaches, and making conclusions based on the pattern of results across approaches.

## Introduction

Does having more money make someone feel happier? The answer to this longstanding question has implications for how individuals live their lives and societies are structured. It is often assumed that more income brings more happiness (with happiness broadly defined herein as hedonic feelings, while recognizing closely related constructs, including satisfaction and eudaimonia; Tiberius, 2006; Angner, 2010; Dolan and Kudrna, 2016; Sunstein, 2021). In many aspects of policy, upward income mobility is encouraged, and poverty can result in exclusion, stigmatization, and discrimination by institutions and members of the public. More income provides people with opportunities and, sometimes, capabilities to consume more and thus satisfy more of their preferences, meet their desires and obtain more of what they want and need (Harsanyi, 1997; Sen, 1999; Nussbaum, 2008). These are all reasons to assume that higher income will bring greater happiness—or, at least, that low income will bring low happiness.

Some research challenges the assumption that earning more should lead to greater happiness. First, because people expect that more money should make them happier, people may feel less happy when their high expectations are not met (Graham and Pettinato, 2002; Nickerson et al., 2003) and they may adapt more quickly to more income than they expect (Aknin et al., 2009; Di Tella et al., 2010). Second, since the 1980s in many developed countries, the well-educated have had less leisure time than those who are not (Aguiar and Hurst, 2007) and people living in high-earning and well-educated households report feeling more time stress and dissatisfaction with their leisure time (Hamermesh and Lee, 2007; Nikolaev, 2018). The quantity of leisure time is not linearly related to happiness, with both too much and too little having a negative association (Sharif et al., 2021). Evidence also shows that people with higher incomes spend more time alone (Bianchi and Vohs, 2016). The lower quality and quantity of leisure and social time of people with higher incomes may, in turn, negatively impact their happiness, especially given there are strong links between social capital or “relational goods” and well-being (Helliwell and Putnam, 2004; Becchetti et al., 2008).

At the same time, some—but not all—evidence suggests that working class individuals tend to be more generous and empathetic than more affluent individuals (Kraus et al., 2010; Piff et al., 2010; Balakrishnan et al., 2017; Macchia and Whillans, 2022), and such kindness toward others has been associated with higher well-being (Dunn et al., 2008; Aknin et al., 2012). Relatedly, psychological research suggests that people with lower socioeconomic status have a more interdependent sense of self (Snibbe and Markus, 2005; Stephens et al., 2007). It is, therefore, possible that people high in income have lower well-being because they experience less of the internal “warm glow” (Andreoni, 1990) benefit that comes along with valuing social relationships and group membership. In theory, therefore, there are reasons to suppose that high income has both benefits and costs for well-being, and empirical evidence can inform the debate about when and whether these different perspectives are supported.

### Empirical Evidence on Income and Happiness

The standard finding in existing literature is that higher income predicts greater happiness, but with a declining marginal utility (Dolan et al., 2008; Layard et al., 2008): that is, higher income is most closely associated with happiness among those with the least income and is least closely associated with happiness for those with the most income. Recently, this finding has been qualified by studies showing that the relationship between income and happiness depends on how happiness is conceptualized and measured: as an overall evaluation of one’s life or as daily emotional states (Kahneman and Deaton, 2010; Killingsworth, 2021). In this vein, authors Kushlev et al. (2015) found no relationship between income and daily happiness in the American Time Use Survey (ATUS), which has recently been found for other happiness measures, too (Casinillo et al., 2020, 2021) The finding from Kushlev et al. (2015) was replicated in the German Socioeconomic Panel Survey (GSEOP) by Hudson et al. (2016), and in another analysis of the ATUS by Stone et al. (2018).

Some research has focused specifically on the effect of high income on happiness. Kahneman and Deaton (2010) conducted regression analyses using a Gallup sample of United States residents, finding that annual income beyond ~$75K was not associated with any higher daily emotional well-being. Income beyond ~$75K, however, predicted better life evaluations. Using a self-selecting sample of experiential data in the United States, Killingsworth (2021) conducted piecewise regressions and found no evidence of satiation or turning points. Jebb et al. (2018) fit regression spline models to global Gallup data, showing that the satiation point in daily experiences found by Kahneman and Deaton (2010) was also apparent in other countries. Unlike Kahneman and Deaton (2010), however, Jebb et al. (2018) also found evidence of satiation in people’s life evaluations, and even some evidence for “turning points”—whereby richer people evaluated their lives as worse than some of those with lower incomes. A satiation point in life evaluations was also found in European countries at around €28K annually (Muresan et al., 2020).

This pattern of findings could partly depend on the choice of analytic strategy. In analyses of the same dataset as Jebb et al. (2018) but using lowess regression, researchers found no evidence of satiation or turning points in the relationship between income and people’s life evaluations (Sacks et al., 2012; Stevenson and Wolfers, 2012). These conflicting results suggest that the effect of analytic strategy on results deserves a closer examination.

### The Research Gap

While there has been much research on income and happiness, including according to how happiness is defined and measured, we are not away of any studies that have compared the relationship between income and happiness according to how income is defined and measured. We propose that the relationship between income and happiness may depend not only on how happiness is measured, but also on how income is measured and analyzed. To improve our knowledge of the relationship between income and happiness, this paper, we focus on nonlinearities in the relationship between income and happiness and re-analyze the ATUS data used by Kushlev et al. (2015) and Stone et al. (2018), as well as the GSOEP data used by Hudson et al. (2016). Specifically, while Kushlev et al. (2015) analyzed income as a continuous variable in the ATUS, we treat income the way it was measured: as a categorical variable. We compare these results to GSOEP data where we re-code the original continuous measure of income into categorical quantiles. To further explore nonlinearities in the relationship between income and happiness, we also conduct local linear “lowess” and spline regression analyses.

We chose to re-analyze these data to address the question of differences in the relationship between income and happiness according to the measurement and analysis of income because the ATUS and GSOEP provide nationally representative data on people’s feelings as experienced during specific “episodes” of the day after asking them to reconstruct what they did during the entire day. Thus, compared to data from Gallup, which measures affect “yesterday,” measurements in the ATUS are more grounded in specific experiences, and therefore, less subject to recall bias (Kahneman et al., 2004). And unlike Gallup, which uses more crude, dichotomous (“yes-no”) response scales, ATUS measures happiness along a standard seven-point Likert-type scale. In the GSOEP, we were also able to analyze data from the Experience Sampling Methodology (ESM), which asks people how they are feeling during specific episodes during the day and, as such, is even more grounded in specific experiences.

### Measuring and Analyzing Income

The original ATUS income variable—family income—contains 16 uneven categories (see Table 1). For example, Category 11 has a range of ~$10K, whereas Category 14 has a range of ~$25K. The increasingly larger categories are designed to reflect declining marginal utility as an innate quality of income. Based on this, Kushlev et al. (2015) analyzed income as a continuous variable using the original uneven categories. Continuous scales, however, assume equal intervals between scale points—a strong assumption to make for the relatively arbitrary rate of change in the category ranges. Is increasing one’s income from $20,000 to $25,000 really equidistant to increasing it from $35,000 to $40,000 (Table 1)? And can we really assume, for example, that adding $5,000 of additional income to $35,000 is the same as adding $10,000 of additional income to $40,000? Recognizing this issue, income researchers have adopted alternative strategies. For example, Stone et al. (2018) took the midpoints of each category of income, and then log-transformed it. Thus, they transformed the categorical measure of income into a continuous measure. This approach produced results for happiness consistent with the findings of Kushlev et al. (2015).

**Table 1:** The original categories of income in the ATUS family income measure with number of individuals in each income category in the ATUS 2010, 2012, and 2013 well-being modules.

Group number	Income range	*N* (individuals)
1	Less than $5,000	883
2	$5,000–$7,499	645
3	$7,500–$9,999	903
4	$10,000–$12,499	1,221
5	$12,500–$14,999	1,096
6	$15,000–$19,999	1,773
7	$20,000–$24,999	2,005
8	$25,000–$29,999	1,989
9	$30,000–$34,999	2,044
10	$35,000–$39,999	1,809
11	$40,000–$49,999	2,959
12	$50,000–$59,999	2,831
13	$60,000–$74,999	3,466
14	$75,000–$99,999	4,011
15	$100,000–$149,999	3,706
16	$150,000 and over	2,635

Complete cases only for all variables analyzed.

Both the increasing ranges of the income scale itself and its log-transformations reflect an assumed declining marginal utility of income: They treat a given amount of income increase at the higher end of the income distribution as having less utility than the same amount at the lower end of the distribution. But by subsuming income’s declining utility in its very measurement (or transformation thereof), it becomes difficult to interpret a null relationship with happiness. In other words, we might not be seeing a declining marginal utility of income reflected on happiness because the income variable itself reflects its declining utility.

Even when the income variable itself does not reflect its declining utility, a null relationship between income and daily experiences of happiness has been observed. Hudson et al. (2016) used GSOEP, which contains a measure of income that is continuous in its original form. Whether analyzing this income measure in its raw original form or in transformed log and quadratic forms, a null relationship with happiness was observed. This approach, however, does not consider whether there might be nonlinear/log/quadratic turning or satiation points at higher levels of income—an issue also applicable to previous analyses of ATUS (Kushlev et al., 2015; Stone et al., 2018). This is important because there are theoretically both benefits and costs to achieving higher levels of income that could occur at various levels of income; however, this possibility has not yet been fully explored in ATUS or GSOEP data.

In sum, past research using ATUS has treated categorically measured income as a continuous variable, either assuming equidistance between scale points or attempting to create equidistance through statistical transformations. By doing so, however, researchers may have statistically accounted for the very utility of income for happiness that they are trying to test. In both ATUS and GSOEP, the question of whether there might be satiation and/or turning points at higher levels of income has not been fully considered. The present research explores whether treating income as a categorical variable in both ATUS and GSOEP would replicate past findings or reveal novel insights, focusing on possible nonlinearities in the relationship between income and happiness.

## Materials and Methods

### Samples

We used data from ATUS well-being modules in 2010, 2012, and 2013. To facilitate future replications of this research, the ATUS extract builder was used to create the dataset (Hofferth et al., 2017).^1^ The ATUS is a repeated cross-sectional survey and is nationally representative of United States household residents aged 15 years and older. Its sampling frame is the Current Population Survey (CPS), which was conducted 2–5 months prior to the ATUS. Some items in the ATUS come from the CPS, including the household income item that we analyze.

Data from the GSOEP come from the Innovation Sample (IS), which is a subsample of the larger main GSOEP (Richter and Schupp, 2015). The main GSOEP and the IS are designed to be nationally representative. The IS contains information on household residents aged 17 years of age and older. We used two modules from these data: the 2012–2015 DRM module, which is a longitudinal survey, and the 2014–2015 ESM module.

### Outcome Measures

In ATUS, participants were called on the phone and asked how they spent their time yesterday: what activities they were doing, for how long, who they spent time with and where they were located. This information was used to create their time use diary. A random selection of three activities were taken from these diaries and participants were asked how they felt during them. The feelings items were tired, sad, stressed, pain, and happy. Participants were also asked how meaningful what they were doing felt.

In GSOEP, participants were interviewed face to face for the DRM questions and through smartphones for the ESM questions. In the DRM, as in the ATUS, they were asked how they spent their time yesterday and, for a random selection of three activities, they were asked further details about how they felt. In the ESM, participants were randomly notified on mobile phones at seven random points during the day for around 1 week. As in the DRM, they were asked how they were spending their time at the point of notification, as well as how they felt. Participants in both ESM and DRM samples were asked about whether they were feeling happy, as well as other emotions such as sadness, stress, and boredom.

The focus of this research is on the happiness items from both the ATUS and GSOEP to highlight differences according to the treatment of the independent measure of income rather than differences according to the dependent outcome of emotional well-being.

### Analyses

Data were analyzed in STATA 15 and jamovi. The Supplementary Material S1 file contains the STATA command file for the main commands written to analyze the data. In both ATUS and GSOEP, OLS regressions were conducted with happiness as the outcome measure and income as the explanatory measure. Following Kushlev et al. (2015) and Hudson et al. (2016), the average happiness across all activities each day was taken to create an individual-level measure. Because the GSOEP DRM sample contained multiple observations across years, the SEs were clustered at the individual level for models using this dataset.

The treatment of income differed according to the dataset because income was collected differently in each dataset. In the ATUS, income was first analyzed in continuous, log, and quadratic forms in OLS regressions, as in other research (Kushlev et al., 2015; Hudson et al., 2016). Next, it was analyzed as a categorical variable with 16 categories, preserving the identical format that it was originally collected in from the CPS questionnaire.

In GSOEP, the income variable in the dataset is provided in continuous form because participants reported their monthly income as an integer. To compare to the ATUS results, 16 quantiles of income were created and analyzed in GSOEP DRMs (see Table 2 - note that there were insufficient observations to conduct these analyses with GSOEP ESMs). This income variable was also analyzed in continuous, log, and quadratic forms.

**Table 2:** The range and number of person-year observations of the GSOEP Income 4 variable divided into 16 quantiles.

Quantile number	Income minimum	Income maximum	*N* (observations)
1	2,400	11,520	433
2	11,616	14,400	459
3	14,472	18,000	584
4	18,024	19,200	228
5	19,356	21,600	427
6	21,840	24,000	520
7	24,120	26,880	306
8	26,940	30,000	660
9	30,240	32,400	257
10	33,000	36,000	631
11	36,360	38,400	193
12	39,000	42,000	430
13	42,600	48,000	539
14	49,032	54,000	289
15	54,720	64,800	400
16	66,000	360,000	410

Complete cases only for all variables analyzed.

Omnibus *F*-tests and effect sizes (*n*^2^) are also reported to compare the categorical, continuous, log, and quadratic approaches.

We conducted lowess and spline regressions to further investigate possible nonlinearities in the relationship between income and happiness. For the lowess regressions, the smoothing parameter was set at of 0.08. For the regression splines, we fitted knots at four quartiles and five quantiles of income. We also used the results of OLS regressions treating income as a categorical variable, as well as the results of the lowess regression treating income as continuous, to fit knots at pre-specified values of income (where these analyses suggested there could be turning and/or satiation points).

Complete case analyses were conducted with 33,976 individuals in ATUS, 6,766 individuals in German DRMs, and 249 individuals in German ESMs. There was item-missing data in some samples (ATUS, 1.7% missing; GSOEP DRMs, 8.2% missing; GSOEP ESMs data, and 6.0% missing). We make analytical and not population inferences and therefore do not use survey weights (Pfeffermann, 1996).

### Controls

Results are presented without and with controls for demographic and diary characteristics. Following Kushlev et al. (2015), Hudson et al. (2016), and Stone et al. (2018), these controls were age, gender, marital status, ethnic background,^2^ health,^3^ employment status, children,^4^ and whether the day was a weekend. We also control for the year of the survey in ATUS DRM data to address the issue that our results are not due to new data but rather how we treat the income variable.

The list of variables we use in analyses are in Table 3.

**Table 3:** List of variables used in analyses in ATUS and GSOEP.

Variable	ATUS	GSOEP
Happiness	x	x
Income		
*Continuous*	x	x
*Log*	x	x
*Quadratic*	x	x
*Categorical*	x	x
Age	x	x
Gender	x	x
Marital status	x	x
Ethnic background		
*Hispanic/Black*	x	
*German origin*		x
Health		
*Physical or cognitive difficulty*	x	
*Self-rated general health*		x
Employment status	x	x
Children		
*Children <18 years in household*	x	
*Number of children*		x
Diary day was weekend	x	x
Year of survey	x	

## Results

In both ATUS and GSOEP, daily happiness was analyzed using a 0–6 scale (in GSOEP scale points 1–7 were recoded to 0–6 to match ATUS). The ATUS mean happiness was 4.38 (SD = 1.33). The GSOEP DRM mean happiness was 2.91 (SD = 1.46), and the GSOEP ESM mean happiness was 2.65 (SD = 1.03).

### Magnitude

The magnitude of our results can be considered in the context of effect sizes from other research on demographic characteristics and daily happiness (Kahneman et al., 2004; Stone et al., 2010; Luhmann et al., 2012; Hudson et al., 2019). For example, the effect size for the relationship between age and daily experiences of happiness was 0.16 in Stone et al. (2010). Our effect sizes range from 0.06 to 0.37. Throughout, we focus on coefficients, their 95% CIs, and visualizations of these coefficients and CIs, rather than on their statistical significance (Lakens, 2021). The purpose of this is to highlight how analytic treatments of income affect the magnitude and precision of the relationship between income and happiness.

### ATUS-DRM

When treating the 16-category family income variable as continuous in OLS regressions, there was no substantive relationship between income and happiness as in other prior research (Kushlev et al., 2015; Hudson et al., 2016; Stone et al., 2018). Out of the linear, squared, and log coefficients without and with controls, the largest and most precise coefficients were with controls; for linear income it was (*b* = −0.006, 95% CI = −0.01, −0.002), squared income (*b* = −0.0001, 95% CI = 0.0003, 0.00006), and log income (*b* = −0.03, 95% CI = −0.05, 0.001). The omnibus *F*-test (without controls) for linear income was *F* = 0.28, *n*^2^ = 0.000008 (95% CI = 0.00, 0.0002), for income squared was *F* = 1.60, *n*^2^ = 0.00005 (95% CI = 0.00, 0.0003), and for log income was *F* = 0.23, *n*^2^ = 0.000006 (95% CI = 0.00,0.0002).

**Figure 1:**
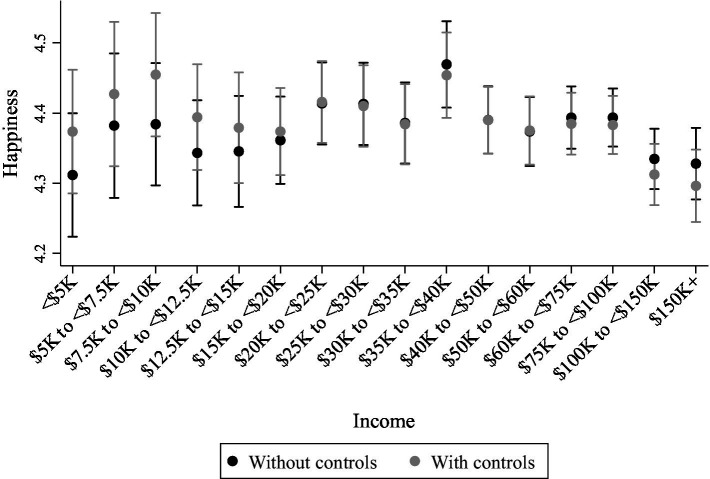
Predicted values of average individual happiness in the American Time Use Survey (ATUS) at the 16 values of the family income variable without and with controls. Covariates at means. 95% CI.

The categorization of income focused attention on those with incomes of $35–40K, who appeared substantively happier than some of those with higher incomes (and lower incomes; see Figure 1). For example, with controls, those with incomes of $35–40K appeared happier relative to those with incomes of $150K+ (*b* = 0.16, 95% CI: 0.08, 0.24) and $100–150K (*b* = 0.14, 95% CI: 0.07, 0.221). The omnibus test for categorical income was *F* = 1.61, *n*^2^ = 0.007 (95% CI = 0.00, 0.0009).

**Figure 2:**
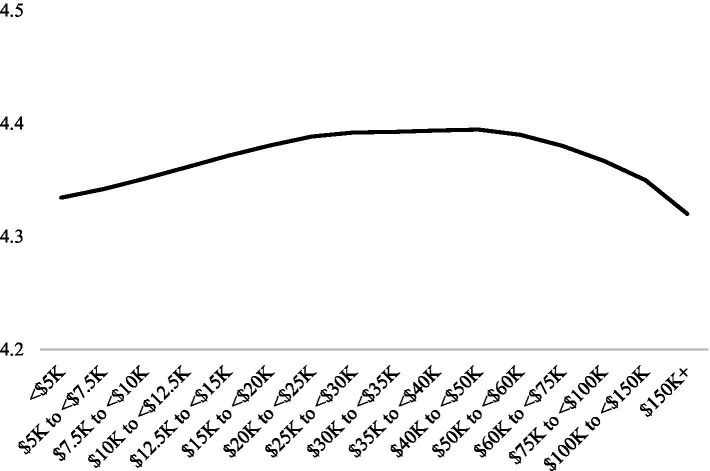
Line graph of predicted values from lowess regressions explaining variance in happiness from income treated as a continuous variable in ATUS.

Results from regression splines and a lowess regression suggested null results overall (see Figure 2). Further details of the analyses are in Supplementary Material S2.

### GSOEP-DRM

When treating the continuous household income variable as continuous (in €10,000s) in OLS regressions, there was no substantive relationship between income and happiness as in other prior research (Kushlev et al., 2015; Hudson et al., 2016; Stone et al., 2018). The association with the largest magnitude and most precision was for log income with controls (*b* = −0.08, 95% CI = −0.18, 0.01).^5^

**Figure 3:**
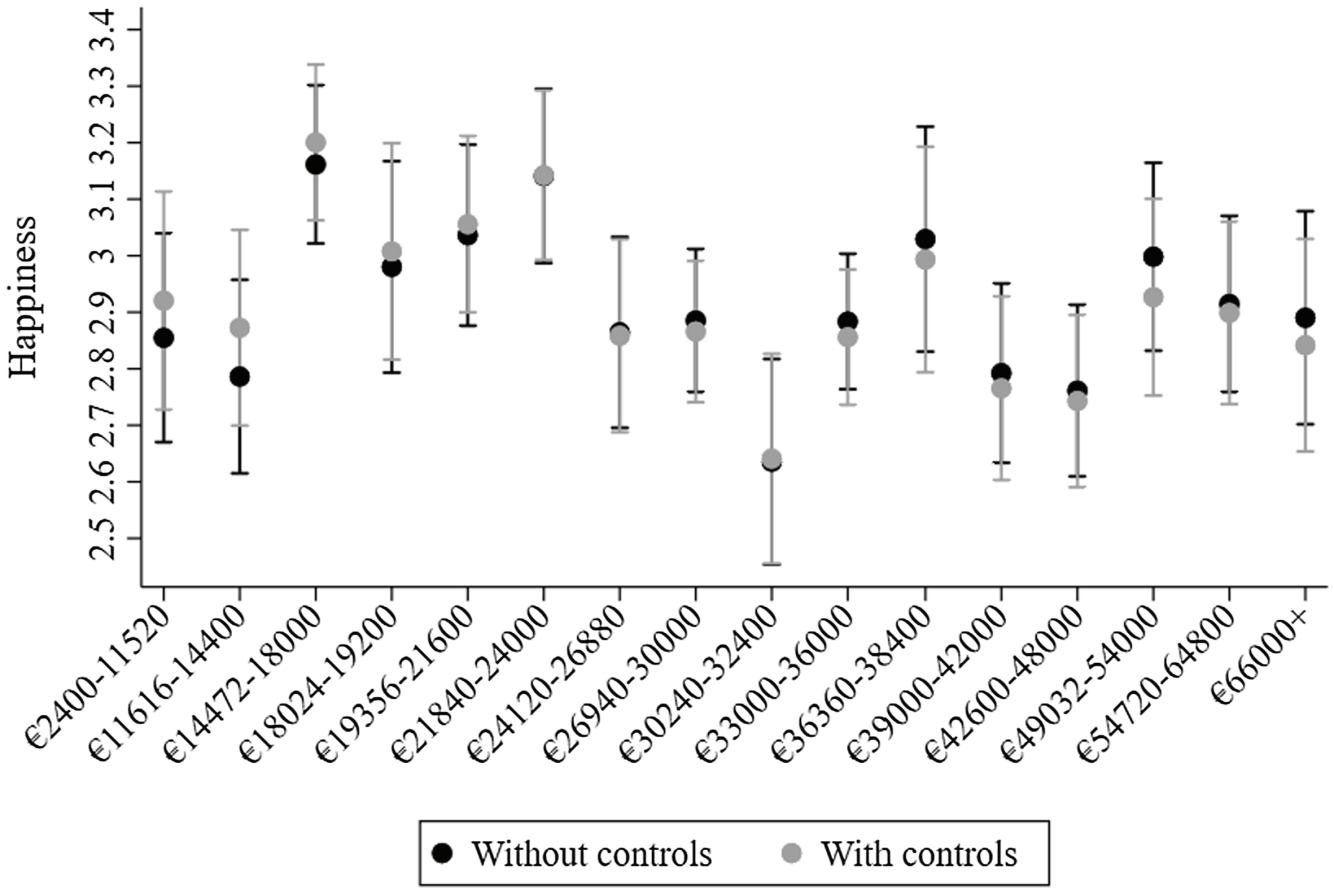
Predicted values of average person-year happiness from GSOEP DRMs at 16 quantiles of income (Income 4) without and with controls. Covariates at means. 95% CI.

As in ATUS, treating the variable as categorical suggested some relationships between income and happiness. These results drew attention to those third quantile (~€14–18K), who seemed happier than those both higher and lower in income (see Figure 3). For example, with controls, they were happier than those in quantiles 13 (€42.6–48K, *b* = 0.46, 95% CI = 0.25, 0.67), seven (~€24–27K, *b* = 0.34, 95% CI = 0.13, 0.56), and one (€2.40–11,520K, *b* = 0.28, 95% CI = 0.05, 0.51). The omnibus test for categorical income was *F* = 4.00, *n*^2^ = 0.009 (95% CI = 0.003, 0.01), whereas the omnibus test for linear income was *F* = 0.09, *n*^2^ = 0.00001 (95% CI = 0.00, 0.0007). The omnibus for log income was *F* = 1.42, *n*^2^ = 0.0002 (95% CI = 0.00, 0.0001) and for income squared it was *F* = 0.96, *n*^2^ = 0.0001 (95% CI = 0.00, 0.001).

**Figure 4:**
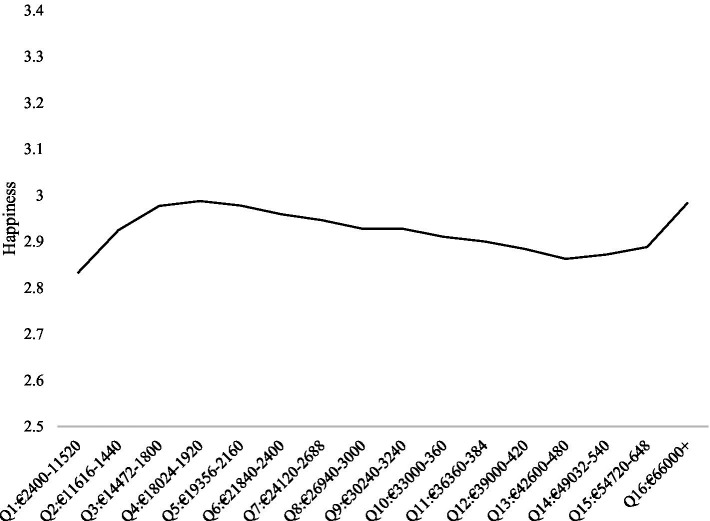
Line graph of predicted values from lowess regressions explaining variance in happiness from income treated as a continuous variable in GSOEP DRMs at 16 quantiles of income.

The lowess and spline regressions suggested null results overall, as the coefficients were small in magnitude (see Figure 4). Further details of the analyses are in Supplementary Material S3.

### GSOEP-ESM

**Figure 5:**
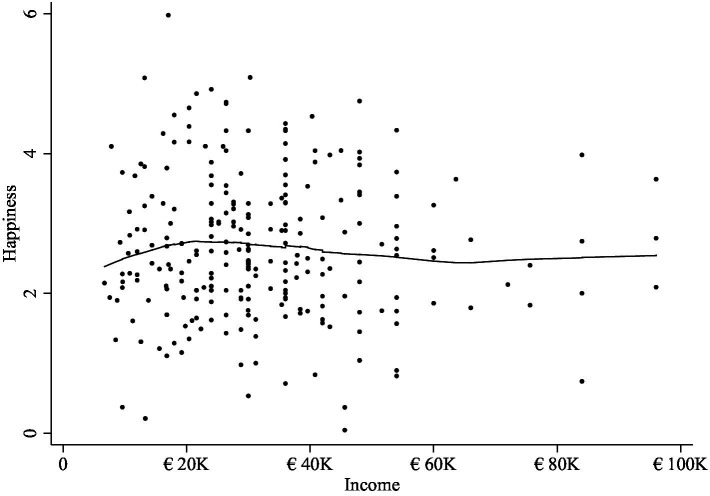
Results of local linear “lowess” regression from GSOEP Experience Sampling Methodology (ESM) data with happiness as the outcome and continuous annual income as the explanatory variable.

There was no evidence to suggest any substantive association between income and happiness in ESM data for linear income, income squared, log income, in the lowess regressions, or regression splines. A visualization of the lowess results are in Figure 5 and further details of the analyses are in Supplementary Material S4.

The omnibus *F*-test for linear income was *F* = 0.53, *n*^2^ = 0.002 (95%CI = −0.00, 0.03), and for log income it was *F* = 0.12, *n*^2^ = 0.0005, 95%CI = 0.00, 0.02. For income squared it was *F* = 0.63, *n*^2^ = 0.003, 95%CI = 0.00 0.03.

## Discussion

Is income creating a signal in these data on daily experiences of happiness, or is it all simply noise? The present results suggest that whether income can be concluded as being associated with daily experiences of happiness may depend on how income is analyzed. When income in ATUS is analyzed in its original, categorical form, there is some evidence that some people with higher incomes feel somewhat less happy than some of those with lower incomes. When the continuous income variable in GSOEP is split into categories, a similar pattern is observed. This is not inconsistent with the findings of Kushlev et al. (2015), Hudson et al. (2016), and Stone et al. (2018), who found no relationship between income and daily feelings of happiness in the same data when income was analyzed as a continuous variable. It simply illustrates that a relationship between income and happiness could be interpreted when treating income categorically rather than continuously.

There are at least three possible interpretations to our overall results. One interpretation tends toward conservative. We conducted multiple comparisons of many transformations of income, which might inspire some to question whether we should have accounted for this in some way by adjusting for multiple comparisons. Although we found some evidence of differences in happiness according to income, such an adjustment might lead to an overall null conclusion when characterizing the relationship between income on happiness. A second interpretation is more generous. Within this perspective, one might emphasize the fact that because our income measures were correlated, no correction for multiple comparisons was required. It could then be argued that because we found some evidence for the relationship between income on happiness, there is good evidence that the overall effect is not null. A more moderate perspective, and the one adopted in this paper, is that because the overall pattern of our results showed mixed null and nonnull results, we can make an overall conclusion of some differences in happiness according to income. We also noticed that equivalizing income in the German data strengthened the relationship of income and happiness, further supporting the conclusion of some differences—and that the analytic treatment of income matters.

Based on the moderate perspective, we conclude that there is very little evidence of any relationship between income and daily experiences of happiness—and any relationship that does exist would suggest higher income could be associated with less happiness. The results do not support the results of Sacks et al. (2012) or Killingsworth (2021), where a greater income was associated with greater happiness, and there were no satiation or turning points (see also Stevenson and Wolfers, 2012). These results are more aligned with Kahneman and Deaton (2010), who found a satiation point in the relationship between income daily experiences of happiness, researchers finding no association between income and happiness (Kushlev et al., 2015; Jebb et al., 2018; Casinillo et al., 2020, 2021), who found that higher income can be associated with worse evaluations of life. We suggest the analytic strategy for income could contribute to explaining discrepant results in existing literature, and researchers should be clear about the approaches they have tested, although we acknowledge that sampling differences could play a role, too.

Overall, the results were broadly consistent between countries because there was no substantive relationship between income and happiness when income was treated continuously but there appeared to be relationships when treating income categorically. Despite a similar overall pattern in the income results, there were other difference between countries. German residents rated their happiness as lower than United States residents (a difference of ~1.5 scale points out of seven). This could be because of different interpretations of the word “happiness” in Germany and the United States. The word for happiness in German used in the survey—*glück*—can mean something more akin to lucky or optimistic—which is different from the meaning of word “happy” in the United States. Despite this linguistic difference, those with higher incomes were still less happy than some of those with lower incomes in both samples.

### Limitations

One limitation to our results is the representativeness of the income distribution. Household surveys like those that we used do not tend to capture the “tails” of the income distribution very well: People in institutions and without addresses are excluded from these sample populations, which omits populations such as those living in nursing homes and prisons, as well as the homeless. Moreover, people do not always self-report their income accurately due to issues such as social desirability bias (Angel et al., 2019). Existing studies that have focused on those with very low incomes do tend to find that low income is associated with low happiness (Diener and Biswas-Diener, 2002; Clark et al., 2016; Adesanya et al., 2017). In ATUS, the highest household income value available was $150K, whereas in GSOEP it was €360K. Thus, it is not always clear whether the very affluent, such as millionaires, are represented in these samples (Smeets et al., 2020). Overall, our results cannot be taken as representative of people who are very poor or rich and should not be interpreted as such.

Another limitation is that the present results cannot be interpreted casually because there has been no manipulation of income in these data nor exploration of mechanisms and there was no longitudinal data in ATUS. As discussed by Kushlev et al. (2015), there are issues such as reverse causality. Here, however, some of our results potentially suggest an alternative reverse causality pathway, whereby less happy people may select into earning more income. Because the counterfactual is not apparent—we do not know how happy people with high incomes would be without their higher income—it could also be that those with high incomes would be even less happy than they currently are if they had not attained their current level of income. In other words, people with high incomes may have started out as less happy in the first place and be even less happy if they did not have high incomes.

A further limitation is the time period of the data, especially that they were collected prior to the COVID-19 pandemic. This could be an issue because it is possible that the relationship between income and daily experiences of happiness has changed, such as due to the exacerbation of health inequalities and restrictions on freedom of movement due to nationwide lockdowns. Our study does not provide any information on the longer-term and health and well-being consequences of both COVID-19 itself and the policy response to COVID-19 (Aknin et al., 2022). As one example, access to green space, which has health and well-being benefits, is lower among those with low income, and this mechanism between income and happiness may have become more salient during COVID-19 (Geary et al., 2021). Overall, it is important to consider the regional, political, and socioeconomic contexts in which income is attained to understand its relationship with well-being, including levels of income in reference groups such as neighbors, friends, and colleagues (Luttmer, 2005; De Neve and Sachs, 2020). It would be important to replicate the results in this research with more recent data to address the limitation that the data we used are not recent, considering our broader point that the measurement and analysis of income should be considered as carefully as the measurement and analysis of happiness.

### Future Directions

This research points to several directions for future research. One direction relates to data and measures: Nonlinearities in the relationship between income and happiness could be examined using time use data from other countries, considered between countries and/or within countries over time (Deaton et al., 2008; De Neve et al., 2018), and investigated for measures of emotional states other than happiness (Piff and Moskowitz, 2018). In general, our results suggest that researchers should pay attention to how income is measured and analyzed when considering how it is related to happiness, which complements findings from other research that the way happiness is measured and analyzed is important (Kahneman and Deaton, 2010; Jebb et al., 2018).

Future research could also explore mechanisms that may explain our findings. In addition to those mentioned in the Introduction—expectations (Graham and Pettinato, 2002; Nickerson et al., 2003), time use (Aguiar and Hurst, 2007; Hamermesh and Lee, 2007; Bianchi and Vohs, 2016; Nikolaev, 2018; Sharif et al., 2021); generosity (Dunn et al., 2008; Kraus et al., 2010; Piff et al., 2010; Aknin et al., 2012; Balakrishnan et al., 2017; Macchia and Whillans, 2022), and sense of self (Snibbe and Markus, 2005; Stephens et al., 2007)—another is the identity-related effect of transitioning between socioeconomic groups. Though one might expect upward mobility to be associated with greater happiness, research suggests that some working class people do not wish to become upwardly mobile because it could lead to a loss of identity and change in community (Akerlof, 1997; Friedman, 2014). Indeed, upward intergenerational mobility is associated with worse life evaluations in the United Kingdom—though not in Switzerland (Hadjar and Samuel, 2015), although recent findings show substantial negative effects of downward mobility, too (Dolan and Lordan, 2021). Over time, therefore, the degree of mobility in a population could influence the relationship between income and happiness in both positive and negative directions.

Additionally, social comparisons could drive the effects of higher income on happiness. Higher income might not benefit happiness if one’s reference group—that is, the people to whom we compare or have knowledge of in some form (Hyman, 1942; Shibutani, 1955; Runciman, 1966)—changes with higher socioeconomic status. As income increases, people might compare themselves to others who are also doing similarly or better to them, and then not feel or think that they are doing any better by comparison—or even feel worse (Cheung and Lucas, 2016). This is one of the explanations for the well-known “Easterlin Paradox” (Easterlin, 1974), which suggests that as national income rises people do not become happier because they compare their achievements to others. The paradox is debated (Sacks et al., 2012). Additionally, some research shows that it is possible to view others’ greater success as one’s own future opportunity and for upward social comparisons to then positively impact upon well-being (Senik, 2004; Davis and Wu, 2014; Ifcher et al., 2018). As with the role of mobility in the relationship between income and happiness, it is unclear whether the role of social comparisons would create a positive or negative impact over time and future research could explore this.

### Final Remarks

Overall, our results provide some evidence that individual attainment in terms of income may not equate to the attainment of individual happiness—and could even be associated with less daily happiness, depending upon how income is measured and analyzed. These results suggest that how income is associated with happiness depends on how income is measured and analyzed. They provide some support to the idea that financial achievement can have both costs and benefits, potentially informing normative discussions about the optimal distribution of income in society.

## Data Availability Statement

Publicly available datasets were analyzed in this study. These data can be found at: https://www.atusdata.org (The ATUS extract builder was used to create the ATUS dataset, see Hofferth et al., 2017). GSOEP data were requested from https://www.diw.de/en/diw_02.c.222516.en/data.html, see Richter and Schupp, 2015.

## Ethics Statement

Ethical review and approval was not required for the study on human participants in accordance with the local legislation and institutional requirements. Written informed consent from the participants’ legal guardian/next of kin was not required to participate in this study in accordance with the national legislation and the institutional requirements.

## Author Contributions

LK and KK contributed to conception and design of the study. LK organized the data, performed the statistical analysis in STATA, and wrote the first draft of the manuscript. KK performed additional statistical analysis in jamovi and wrote sections of the manuscript. All authors contributed to the article and approved the submitted version.

## Funding

LK was supported by a London School of Economics PhD scholarship during early work and later by the National Institute for Health Research (NIHR) Applied Research Collaboration (ARC) West Midlands. The views expressed are those of the author(s) and not necessarily those of the NIHR or the Department of Health and Social Care.

## Conflict of Interest

The authors declare that the research was conducted in the absence of any commercial or financial relationships that could be construed as a potential conflict of interest.

## Publisher’s Note

All claims expressed in this article are solely those of the authors and do not necessarily represent those of their affiliated organizations, or those of the publisher, the editors and the reviewers. Any product that may be evaluated in this article, or claim that may be made by its manufacturer, is not guaranteed or endorsed by the publisher.

## Footnotes

^1^ https://www.atusdata.org

^2^In the ATUS this was Hispanic and Black, in GSOEP this was German origin.

^3^In the ATUS this was whether the respondent had any physical or cognitive difficulty (yes/no), in GSOEP this was self-rated general health (bad, poor, satisfactory, good, and very good).

^4^In the ATUS this was presence of children <18 years in the household, in GSOEP this was number of children.

^5^This association was stronger and more precise when equivalizing income (dividing by the square root of household size), *b* = −0.16, 95%CI = −0.06, −0.27, underscoring the importance of transparency in the treatment of income.
