# Supplementary material for: Money Does Not Always Buy Happiness, but Are Richer People Less Happy in Their Daily Lives? It Depends on How You Analyze Income
Source: Front Psychol. 2022 May 31;13:883137. doi: 10.3389/fpsyg.2022.883137 (PMC9199446; doi:10.3389/fpsyg.2022.883137)
Supplement: Supplementary file 1 [file Data_Sheet_1.docx]

**S1 File. STATA do file.**

************************************************************************************************************************************************************************************************************************************

************************************************************************************************************************************************************************************************************************************

************************************************************************************************************************************************************************************************************************************

*********************************************************ATUS

************************************************************************************************************************************************************************************************************************************

************************************************************************************************************************************************************************************************************************************

************************************************************************************************************************************************************************************************************************************

******************************************************************************************************************

******************************************************************************************************************

*********************************************************CLEANING

******************************************************************************************************************

******************************************************************************************************************

clear

use "atus extract file.dta"

**happy

gen happy = schappy

replace happy = . if schappy > 7

egen avhappy = mean(happy), by(caseid)

**income

gen famincome2 = 1 if famincome < 12

replace famincome2 = 2 if famincome > 11 & famincome < 15

replace famincome2 = 3 if famincome ==15

replace famincome2 = 4 if famincome ==16

replace famincome2 = . if famincome > 16

label define inclab6 1 "less than $50K" 2 "$50K to less than $100K" 3 "$100K to less than $150K" 4 "$150K+"

label values famincome2 inclab6

**gender

gen gender = sex

label define genlab 1 "male" 2 "female"

label values gender genlab

**marital status

gen married = 1 if marst == 1 | marst == 2

replace married = 2 if marst == 3

replace married = 3 if marst == 4

replace married = 4 if marst == 5

replace married = 5 if marst == 6

label define marlab 1 "married" 2 "widowed" 3 "divorced" 4 "separated" 5 "never married"

label values married marlab

replace married = 0 if married != 1

replace married = . if marst == .

label define marlab2 1 "married" 0 "not married"

label values married marlab2

**education

gen education = 0 if educ == 10 | educ == 11 | educ == 12 | educ == 13 | educ == 14 | educ == 15 | educ == 16 | educ == 17

replace education = 1 if educ == 20 | educ == 21

replace education = 2 if educ == 30

replace education = 3 if educ == 31 | educ == 32

replace education = 4 if educ == 40

replace education = 5 if educ == 41 | educ == 42 | educ == 43

label define edlabel4 0 "<=12th grade (no diploma)" 1 "High school diploma or equiv." 2 "Some college but no degree" 3 "Associate degree" 4 "Bachelor's degree" 5 "MSc/PhD+"

label values education edlabel4

**ethnicity

gen hispanic = 1 if hispan != 100

replace hispanic = 0 if hispan ==100

gen black = 1 if race == 110

replace black = 0 if race != 110

**disability

gen disability = diffany

label define dislab 1 "No" 2 "Yes"

label values disability dislab

**employed

gen employed = 1 if empstat == 1 | empstat == 2

replace employed = 0 if empstat != 1 & empstat !=2

label define emplab 0 "no" 1 "yes"

label values employed emplab

**children

gen children = hh_child_cps8

label define kidslab 0 "no" 1 "yes"

label values children emplab

**weekend

gen weekend = 1 if day == 1 | day == 7

replace weekend = 0 if weekend != 1

label define weekendlab 0 "no" 1 "yes"

label values weekend weekendlab

**unemployed

gen unem = 1 if empstat == 3 | empstat == 4

replace unem = 0 if unem ! = 1

**original income

label define newinclab3 1 "<$5K" 2 "$5K to <$7.5K" 3 "$7.5K to <$10K" 4 "$10K to <$12.5K" 5 "$12.5K to <$15K" 6 "$15K to <$20K" 7 "$20K to <$25K" 8 "$25K to <$30K" 9 "$30K to <$35K" 10 "$35K to <$40K" 11 "$40K to <$50K" 12 "$50K to <$60K" 13 "$60K to <$75K" 14 "$75K to <$100K" 15 "$100K to <$150K" 16 "$150K+"

gen famincnew = famincome

replace famincnew = . if famincome > 17

label values famincnew newinclab3

**income squared

gen famincnewsq = famincnew*famincnew

**log income

gen famincnewlog = log(famincnew)

**making dataset individual (no individuals participated longitudinally)

sort caseid

by caseid: generate n1 = _n

keep if n1==1

**keeping only wellbeing module, number of missing

keep if wb_resp==1

misstable summarize avhappy famincnew age gender married hispanic black disability employed children weekend year

codebook caseid if avhappy == . | famincnew == .

codebook caseid

**saving full dataset

save "atusfull.dta", replace

clear

use "atusfull.dta"

**dropping missing

keep if avhappy != . & famincome != .

**average happiness

mean avhappy

estat sd

**Table 1

tab famincnew

******************************************************************************************************************

******************************************************************************************************************

*********************************************************ANALYSES

******************************************************************************************************************

******************************************************************************************************************

**Continuous income

reg avhappy famincnew

reg avhappy famincnew age i.gender i.married i.hispanic i.black i.disability i.employed i.children i.weekend i.year

**Squared income

reg avhappy famincnewsq

reg avhappy famincnewsq age i.gender i.married i.hispanic i.black i.disability i.employed i.children i.weekend i.year

**Log income

reg avhappy famincnewlog

reg avhappy famincnewlog age i.gender i.married i.hispanic i.black i.disability i.employed i.children i.weekend i.year

**16 categories - relative to category 16

est clear

eststo: reg avhappy ib16.famincnew

margins i.famincnew, saving(file1, replace)

eststo: reg avhappy ib16.famincnew age i.gender i.married i.hispanic i.black i.disability i.employed i.children i.weekend i.year

margins i.famincnew, saving(file2, replace)

combomarginsplot file1 file2, title(" ",) recast(scatter) plot1opts(mcolor(gs0)) plot2opts(mcolor(gs10)) ci1opt(lcolor(black)) ci2opt(lcolor(gs10)) xlab(1(1)16, valuelabel alternate labsize(medsmall)) ylab(4.2(.1)4.5, labsize(small) nogrid) xtitle(" " "Income", size(med)) ytitle("Happiness" " ", size(med)) labels("Without controls" "With controls") graphregion(color(white)) bgcolor(white)

graph save graph1, replace

esttab est1 est2 using results.csv, replace label collabel(none) nonumbers not nonotes cells("b(fmt(%10.2g)) se(fmt(%10.2g)) p(fmt(%10.7e))") stats(r2 N)

**16 categories - relative to $35-40K

est clear

eststo: reg avhappy ib10.famincnew

margins i.famincnew, saving(file1, replace)

eststo: reg avhappy ib10.famincnew age i.gender i.married i.hispanic i.black i.disability i.employed i.children i.weekend i.year

margins i.famincnew, saving(file2, replace)

combomarginsplot file1 file2, title(" ",) recast(scatter) plot1opts(mcolor(gs0)) plot2opts(mcolor(gs10)) ci1opt(lcolor(black)) ci2opt(lcolor(gs10)) xlab(1(1)16, valuelabel alternate labsize(medsmall)) ylab(4.2(.1)4.5, labsize(small) nogrid) xtitle(" " "Income", size(med)) ytitle("Happiness" " ", size(med)) labels("Without controls" "With controls") graphregion(color(white)) bgcolor(white)

graph save graph1, replace

esttab est1 est2 using results.csv, replace label collabel(none) nonumbers not nonotes cells("b(fmt(%10.2g)) se(fmt(%10.2g)) p(fmt(%10.7e))") stats(r2 N)

**Loess income

lowess avhappy famincnew, gen(yhat)

table famincnew, c(mean yhat)

**Splines

**linear quartile

mkspline familyincome 4 = famincnew, pctile displayknots

reg avhappy familyincome1-familyincome4

reg avhappy familyincome1-familyincome4 age i.gender i.married i.hispanic i.black i.disability i.employed i.children i.weekend i.year

**linear quantile

mkspline familyincome_ 5 = famincnew, pctile displayknots

reg avhappy familyincome_1-familyincome_5

reg avhappy familyincome_1-familyincome_5 age i.gender i.married i.hispanic i.black i.disability i.employed i.children i.weekend i.year

**linear specified

mkspline faminclin1 10 faminclin2 = famincnew

reg avhappy faminclin1 faminclin2

reg avhappy faminclin1 faminclin2 age i.gender i.married i.hispanic i.black i.disability i.employed i.children i.weekend i.year

************************************************************************************************************************************************************************************************************************************

************************************************************************************************************************************************************************************************************************************

************************************************************************************************************************************************************************************************************************************

*********************************************************GSOEP DRM

************************************************************************************************************************************************************************************************************************************

************************************************************************************************************************************************************************************************************************************

************************************************************************************************************************************************************************************************************************************

******************************************************************************************************************

******************************************************************************************************************

*********************************************************MERGING

******************************************************************************************************************

******************************************************************************************************************

clear

use "idrm.dta"

merge m:1 pid syear using "/p.dta"

drop _merge

merge m:1 cid hid syear using "h.dta"

drop _merge

merge m:1 pid cid hid syear using "pgen.dta"

drop _merge

merge m:1 pid using "ppfad.dta"

drop _merge

merge m:1 pid syear using "phrf.dta"

save "gsoep merged.dta", replace

clear

use "gsoep merged.dta"

******************************************************************************************************************

******************************************************************************************************************

*********************************************************CLEANING

******************************************************************************************************************

******************************************************************************************************************

clear

use "gsoep merged.dta"

**keeping DRM dataset

keep if im_drm==1

**labeling missing values in happy, sad and income and creating average by person and year

replace emotion1 = . if emotion1 < 1

gen emotion1a = emotion1 -1

egen avhappy = mean(emotion1a), by(pid syear)

codebook pid if avhappy != .

replace emotion5 = . if emotion5 < 1

gen emotion5a = emotion5 -1

egen avsad = mean(emotion5a), by(pid syear)

codebook pid if avsad != .

**determining best variables with none missing for year of birth and gender**

foreach var of varlist gebjahr ple0010 pla0009 sex {

replace `var' = . if `var' <0

}

misstable summarize gebjahr ple0010 pla0009 sex

**age (didn't do monthly as month missing data)*

gen birthyear = gebjahr

replace birthyear = . if gebjahr < 0

gen age = iyear-birthyear

**gender

gen gender = 0 if sex ==1

replace gender = 1 if sex == 2

replace gender = . if sex < 0

label define genlab 0 "male" 1 "female"

label values gender genlab

**married

gen marriedpar = 1 if pld0131 == 1 | pld0131 == 6

replace marriedpar = 0 if pld0131 == 2 | pld0131 == 3 | pld0131 == 4 | pld0131 == 5 | pld0131 == 7

replace marriedpar = . if marriedpar == -2 | pld0131 == -1

gen othermar = pgfamstd

replace othermar = . if pgfamstd < 0

replace marriedpar = othermar if marriedpar ==.

label define marlab 0 "not married/partnered" 1 "married/partnered"

label values marriedpar marlab

**ethnicity / german origin

gen germanborn = germborn

label values germanborn germborn

**health

gen healthsr = . if ple0008 == -1

replace healthsr = 5 if ple0008 == 1

replace healthsr = 4 if ple0008 == 2

replace healthsr = 3 if ple0008 == 3

replace healthsr = 2 if ple0008 == 4

replace healthsr = 1 if ple0008 == 5

label define healthsrlab 1 "Bad" 2 "Poor" 3 "Satisfactory" 4 "Good" 5 "Very good"

label values healthsr healthsrlab

**employment

gen employment = 1 if plb0022 == 3

replace employment = 2 if plb0022 == 5

replace employment = 3 if plb0022 == 9

replace employment = 0 if plb0022 == 1 | plb0022 == 2 | plb0022 == 4 | plb0022 == 6 | plb0022 == 7 | plb0022 == 8

label define emplab 1 "Student (Vocational Training)" 2 "Retired (Near retirement, zero hours)" 3 "Not working (not employed)" 0 "Employed (various)"

label values employment emplab

tab employment

**no. children

gen nokids = hlc0043

replace nokids = . if nokids == -1

replace nokids = 0 if nokids == -2

**weekend

gen weekend = 0 if drm_wt == 2 | drm_wt == 3 | drm_wt == 4 | drm_wt == 5 | drm_wt == 6

replace weekend = 1 if drm_wt == 1 | drm_wt == 7

label define weeklab 0 "Weekday" 1 "Weekend"

label values weekend weeklab

**annual income

gen aninc = hlc0005*12

replace aninc = . if hlc0005==.

replace aninc = . if hlc0005 < 0

**income squared

gen anincsq = aninc*aninc

**log income

gen aninclog = log(aninc)

**16 quantiles of income

xtile pc16aninc = aninc, nq(16)

**making dataset individual

sort pid syear

by pid syear: generate n1 = _n

keep if n1==1

**saving dataset

save "gsoep full.dta", replace

**keeping non-missing values

clear

use "gsoep full.dta"

misstable summarize avhappy age gender marriedpar germanborn healthsr employment nokids weekend iyear

codebook pid if avhappy ==. | aninc ==. | marriedpar ==. | healthsr ==. | nokids ==.

**missing, 604/7370 (person-year), weekend drops anyways

keep if avhappy !=. & age !=. & gender !=. & marriedpar !=. & nokids !=. & germanborn !=. & healthsr !=. & weekend !=. & iyear !=. & employment != .

**Table

table pc16aninc, c(min aninc max aninc n aninc)

**Average happiness

mean avhappy

estat sd

******************************************************************************************************************

******************************************************************************************************************

*********************************************************ANALYSES

******************************************************************************************************************

******************************************************************************************************************

**Continuous income

reg avhappy aninc, vce(cluster pid)

reg avhappy aninc age i.gender i.marriedpar i.nokids i.employment i.germanborn i.healthsr i.weekend , vce(cluster pid)

**Squared income

reg avhappy anincsq, vce(cluster pid)

reg avhappy anincsq age i.gender i.marriedpar i.nokids i.employment i.germanborn i.healthsr i.weekend , vce(cluster pid)

**Log income

reg avhappy aninclog, vce(cluster pid)

reg avhappy aninclog age i.gender i.marriedpar i.nokids i.employment i.germanborn i.healthsr i.weekend , vce(cluster pid)

**16 categories - relative to category 16

est clear

eststo: reg avhappy ib16.pc16aninc, vce(cluster pid)

margins i.pc16aninc, saving(file1, replace)

eststo: reg avhappy ib16.pc16aninc age i.gender i.marriedpar i.nokids i.employment i.germanborn i.healthsr i.weekend , vce(cluster pid)

margins i.pc16aninc, saving(file2, replace)

combomarginsplot file1 file2, title(" ",) recast(scatter) plot1opts(mcolor(gs0)) plot2opts(mcolor(gs10)) ci1opt(lcolor(black)) ci2opt(lcolor(gs10)) xlab(1(1)16, valuelabel labsize(medsmall)) ylab(3.4(.1)2.5, labsize(small) nogrid) xtitle(" " "Income quantile (16)", size(med)) ytitle("Happiness" " ", size(med)) labels("Without controls" "With controls") graphregion(color(white)) bgcolor(white)

graph save graph1, replace

esttab est1 est2 using results.csv, replace collabel(none) nonumbers not nonotes cells("b(fmt(%10.2g)) se(fmt(%10.2g)) p(fmt(%10.7e))") stats(r2 N)

**16 categories - relative to category 3

reg avhappy ib3.pc16aninc , vce(cluster pid)

reg avhappy ib3.pc16aninc age i.gender i.marriedpar i.nokids i.employment i.germanborn i.healthsr i.weekend , vce(cluster pid)

**Loess income

lowess avhappy aninc, gen(yhat)

table pc16aninc, c(mean yhat)

**Splines

**linear quartile

mkspline aninc 4 = aninc, pctile displayknots

reg avhappy aninc1-aninc4 , vce(cluster pid)

reg avhappy aninc1-aninc4 age i.gender i.marriedpar i.nokids i.employment i.germanborn i.healthsr i.weekend , vce(cluster pid)

**linear quantile

mkspline aninc_ 5 = aninc, pctile displayknots

reg avhappy aninc_1-aninc_5 , vce(cluster pid)

reg avhappy aninc_1-aninc_5 age i.gender i.marriedpar i.nokids i.employment i.germanborn i.healthsr i.weekend , vce(cluster pid)

**linear specified

summ aninc if aninc >= 14472 & aninc < = 18000, detail

summ aninc if aninc >= 49032 & aninc < = 54000, detail

mkspline aninclin1 16800 aninclin2 52800 aninclin3 = aninc, displayknots

reg avhappy aninclin1 aninclin2 aninclin3, vce(cluster pid)

reg avhappy aninclin1 aninclin2 aninclin3 age i.gender i.marriedpar i.nokids i.employment i.germanborn i.healthsr i.weekend , vce(cluster pid)

************************************************************************************************************************************************************************************************************************************

************************************************************************************************************************************************************************************************************************************

************************************************************************************************************************************************************************************************************************************

*********************************************************GSOEP ESM

************************************************************************************************************************************************************************************************************************************

************************************************************************************************************************************************************************************************************************************

************************************************************************************************************************************************************************************************************************************

******************************************************************************************************************

******************************************************************************************************************

*********************************************************MERGING

******************************************************************************************************************

******************************************************************************************************************

clear

use "i_esm.dta"

merge m:1 pid syear using "p.dta"

drop _merge

merge m:1 cid hid syear using "h.dta"

drop _merge

merge m:1 pid cid hid syear using "pgen.dta"

drop _merge

merge m:1 pid using "ppfad.dta"

drop _merge

merge m:1 pid syear using "phrf.dta"

save "gsoep ESM merged.dta", replace

******************************************************************************************************************

******************************************************************************************************************

*********************************************************CLEANING

******************************************************************************************************************

******************************************************************************************************************

clear

use "gsoep ESM merged.dta"

**replacing missing values in happy and sad and creating average by person and year

replace q5esm_1 = . if q5esm_1 < 1

gen emotion1a = q5esm_1 -1

egen avhappy = mean(emotion1a), by(pid syear)

codebook pid if avhappy != .

replace hlc0005 = . if hlc0005 < 0

summ hlc0005, detail

**keeping ESM dataset

keep if im_esm==1

**determining best (lowest rate of missing) variables with none missing for year of birth and gender

foreach var of varlist gebjahr ple0010 pla0009 sex {

replace `var' = . if `var' <0

}

misstable summarize gebjahr ple0010 pla0009 sex

**age (didn't do monthly as month missing data)

gen birthyear = gebjahr

replace birthyear = . if gebjahr < 0

gen age = iyear-birthyear

**gender

gen gender = 0 if sex ==1

replace gender = 1 if sex == 2

replace gender = . if sex < 0

label define genlab 0 "male" 1 "female"

label values gender genlab

**married

gen marriedpar = 1 if pld0131 == 1 | pld0131 == 6

replace marriedpar = 0 if pld0131 == 2 | pld0131 == 3 | pld0131 == 4 | pld0131 == 5 | pld0131 == 7

replace marriedpar = . if marriedpar == -2 | pld0131 == -1

gen othermar = pgfamstd

replace othermar = . if pgfamstd < 0

replace marriedpar = othermar if marriedpar ==.

label define marlab 0 "not married/partnered" 1 "married/partnered"

label values marriedpar marlab

**ethnicity / german origin

gen germanborn = germborn

label values germanborn germborn

**health

gen healthsr = . if ple0008 == -1

replace healthsr = 5 if ple0008 == 1

replace healthsr = 4 if ple0008 == 2

replace healthsr = 3 if ple0008 == 3

replace healthsr = 2 if ple0008 == 4

replace healthsr = 1 if ple0008 == 5

label define healthsrlab 1 "Bad" 2 "Poor" 3 "Satisfactory" 4 "Good" 5 "Very good"

label values healthsr healthsrlab

**employment

gen employment = 1 if plb0022 == 3

replace employment = 2 if plb0022 == 5

replace employment = 3 if plb0022 == 9

replace employment = 0 if plb0022 == 1 | plb0022 == 2 | plb0022 == 4 | plb0022 == 6 | plb0022 == 7 | plb0022 == 8

label define emplab 1 "Student (Vocational Training)" 2 "Retired (Near retirement, zero hours)" 3 "Not working (not employed)" 0 "Employed (various)"

label values employment emplab

tab employment

**no. children

gen nokids = hlc0043

replace nokids = . if nokids == -1

replace nokids = 0 if nokids == -2

**annual income

gen aninc = hlc0005*12

replace aninc = . if hlc0005==.

**income squared

gen anincsq = aninc*aninc

**log income

gen aninclog = log(aninc)

**making dataset individual

sort pid syear

by pid syear: generate n1 = _n

keep if n1==1

**weekend

gen dow = dow( mdy( pmonin, ptagin, iyear) )

gen weekend = 0 if dow == 1 | dow == 2 | dow == 3 | dow == 4 | dow == 5

replace weekend = 1 if dow == 0 | dow == 6

label define weeklab 0 "Weekday" 1 "Weekend"

label values weekend weeklab

**some errors missing for one person

replace iyear=2014 if iyear==.

replace weekend = 1 if weekend ==.

save "gsoep ESM full.dta", replace

**Keeping non-missing

clear

use "gsoep ESM full.dta"

***missing values 16/265

misstable summarize phrf employment avhappy aninc age gender marriedpar nokids germanborn healthsr weekend iyear syear

codebook pid if age==. | aninc ==. | marriedpar ==. | healthsr ==. | nokids ==. | employment==.

codebook pid

***dropping missing values

keep if avhappy !=. & aninc !=. & age !=. & gender !=. & marriedpar !=. & nokids !=. & germanborn !=. & healthsr !=. & weekend !=. & iyear !=.

******************************************************************************************************************

******************************************************************************************************************

*********************************************************ANALYSES

******************************************************************************************************************

******************************************************************************************************************

**Continuous income

reg avhappy aninc

reg avhappy aninc age i.gender i.marriedpar i.nokids i.employment i.germanborn i.healthsr i.weekend

**Squared income

reg avhappy anincsq

reg avhappy anincsq age i.gender i.marriedpar i.nokids i.employment i.germanborn i.healthsr i.weekend

**Log income

reg avhappy aninclog

reg avhappy aninclog age i.gender i.marriedpar i.nokids i.employment i.germanborn i.healthsr i.weekend

**Loess income

lowess avhappy aninc, gen(yhat)

**Splines

**linear quartile

mkspline aninc1 19200 aninc2 30000 aninc3 42000 aninc4 = aninc

reg avhappy aninc1-aninc4

reg avhappy aninc1-aninc4 age i.gender i.marriedpar i.nokids i.employment i.germanborn i.healthsr i.weekend

**linear quantile

mkspline aninc_1 18000 aninc_2 25200 aninc_3 34800 aninc_4 4800 aninc_5 = aninc

reg avhappy aninc_1-aninc_5

reg avhappy aninc_1-aninc_5 age i.gender i.marriedpar i.nokids i.employment i.germanborn i.healthsr i.weekend

**linear specified

mkspline aninc1_ 16800 aninc2_ = aninc

reg avhappy aninc1_-aninc2_

reg avhappy aninc1_-aninc2_ age i.gender i.marriedpar i.nokids i.employment i.germanborn i.healthsr i.weekend

mkspline aninc1__ 20000 aninc2__ = aninc

reg avhappy aninc1__-aninc2__

reg avhappy aninc1__-aninc2__ age i.gender i.marriedpar i.nokids i.employment i.germanborn i.healthsr i.weekend

mkspline aninc1___ 52800 aninc2___ = aninc

reg avhappy aninc1___-aninc2___

reg avhappy aninc1___-aninc2___ age i.gender i.marriedpar i.nokids i.employment i.germanborn i.healthsr i.weekend
