# Supplementary material for: Money Does Not Always Buy Happiness, but Are Richer People Less Happy in Their Daily Lives? It Depends on How You Analyze Income
Source: Front Psychol. 2022 May 31;13:883137. doi: 10.3389/fpsyg.2022.883137 (PMC9199446; doi:10.3389/fpsyg.2022.883137)
Supplement: Supplementary file 2 [file Data_Sheet_2.docx]

**S2 File. ATUS DRM results**

*Continuous income (linear, squared, log)*

In regressions that treated the categorical income variable as continuous, there was no substantive relationship between income and happiness both (b=-0.0009, 95% CI=-0.004, 0.003) and with controls (b=-0.006, 95% CI: -0.01, -0.002).

For continuous income squared, again, there was no substantive relationship with happiness without (b=-0.0001, 95% CI: -0.0003, 0.00006) and with controls (b=-0.0004, 95% CI= -0.0006, -0.0001).

The log of income was not substantively associated with happiness without (b=0.006, 95% CI= -0.02, 0.03) and with controls (b=-0.02, 95% CI = -0.05, 0.002).

*Categorical income*

Results of ATUS OLS regressions explaining variance in average happiness according to the 16 categories if income are shown in Fig 1 (main text) and Table 1 S2.

**Table 1 S2. Results of OLS regressions in ATUS explaining variance in average happiness at the individual level from the second (original) family Income 2 variable without and with controls**

|  | **Happy (without controls)** | | | **Happy (with controls)** | | |
| --- | --- | --- | --- | --- | --- | --- |
| *Income 2* | b | 95% CI | | b | 95% CI | |
| <$5K | -0.02 | -0.12, 0.09 |  | 0.08 | -0.03, 0.18 |  |
| $5K to <$7.5K | 0.05 | -0.06, 0.17 |  | 0.13 | 0.01, 0.24 |  |
| $7.5K to <$10K | 0.06 | -0.05, 0.16 |  | 0.16 | 0.05, 0.26 |  |
| $10K to <$12.5K | 0.02 | -0.08, 0.11 |  | 0.1 | 0.00, 0.19 |  |
| $12.5K to <$15K | 0.02 | -0.08, 0.11 |  | 0.08 | -0.02, 0.18 |  |
| $15K to <$20K | 0.03 | -0.05, 0.11 |  | 0.08 | -0.01, 0.16 |  |
| $20K to <$25K | 0.09 | 0.01, 0.16 |  | 0.12 | 0.04, 0.20 |  |
| $25K to <$30K | 0.09 | 0.01, 0.16 |  | 0.11 | 0.03, 0.19 |  |
| $30K to <$35K | 0.06 | -0.02, 0.13 |  | 0.09 | 0.01, 0.16 |  |
| $35K to <$40K | 0.14 | 0.06, 0.22 |  | 0.16 | 0.08, 0.23 |  |
| $40K to <$50K | 0.06 | -0.01, 0.13 |  | 0.09 | 0.02, 0.16 |  |
| $50K to <$60K | 0.05 | -0.03, 0.12 |  | 0.08 | 0.01, 0.15 |  |
| $60K to <$75K | 0.07 | 0.00, 0.13 |  | 0.09 | 0.02, 0.15 |  |
| $75K to <$100K | 0.07 | 0.00, 0.13 |  | 0.09 | 0.02, 0.15 |  |
| $100K to <$150K | 0.01 | -0.06, 0.07 |  | 0.02 | -0.05, 0.08 |  |
| $150K+ | *reference* | | | *reference* | | |
| Constant | 4.32 | 4.28, 4.38 |  | 3.70 | 3.61, 3.78 |  |
| r2 | 0.0007 | | | 0.03 | | |
| N | 33976 | | | 33976 | | |

Without controls, those in the $35-40K income group appeared substantively happier than those with incomes of $150K+ (b=0.14, 95% CI=0.06, 0.22), $100-150K (b=0.13, 95% CI = 0.06, 0.21), $75-100K (b=0.08, 95% CI = 0.001, 0.15), $60-75K (b=0.08, 95% CI = 0.00002, 0.15), $50-60K (b=0.10, 95% CI = 0.02, 0.17), $40-50K (b=0.08, 95% CI = 0.001, 0.15), $15-20K (b=0.11, 95% CI = 0.02, 0.20), $12.5-15K (b=0.12, 95% CI = 0.02, 0.22), $10-12.5K (b=0.13, 95% CI = 0.03, 0.22), and less than $5K (b=0.16, 95% CI = 0.05, 0.26). This group did not significantly differ relative to other income groups.

With controls, those with incomes of $35-40K were only happier relative to those with incomes of $150K+ (b=0.15, 95% CI =0.07, 0.23), $100-150K (b=0.14, 95% CI =0.06, 0.21), and $50-60K (b=-0.08, 95% CI =0.0005, 0.16), whereas differences relative to other income groups were negligible.

*Lowess*

As shown in Fig 2 (main text), there is no evidence of greater happiness associated with greater income after $35-40K in ATUS in lowess regression analyses. There is some evidence of a slight downturn in happiness after this point.

*Splines*

We fit a spline regression with knots at quartiles corresponding to $20-25K, $40-50K and $75-100K. There was no substantive relationship between income and happiness prior to $75-100K but after $75-100K the slope was somewhat negative (b=-0.03, 95% CI = 0.003, 0.06). This result largely held with controls and with quantiles with the highest quantile at $75-100K+). The overall pattern and magnitude suggests a null relationship.

We also fit a spline regression with a pre-specified knot at $35-40K. Without controls, the relationship between income and happiness was small but positive before the knot (b=0.01, 95% CI =0.004, 0.02) and negative after the knot (b=-0.01, 95% CI =-0.02, -0.007). With controls, there was no substantive relationship before the knot (b=0.003, 95% CI =-0.003, 0.01) but the relationship was negative after the knot (b=-0.02, 95% CI =-0.03, -0.008). The overall pattern and magnitude suggest a null relationship.
