# Supplementary material for: Money Does Not Always Buy Happiness, but Are Richer People Less Happy in Their Daily Lives? It Depends on How You Analyze Income
Source: Front Psychol. 2022 May 31;13:883137. doi: 10.3389/fpsyg.2022.883137 (PMC9199446; doi:10.3389/fpsyg.2022.883137)
Supplement: Supplementary file 3 [file Data_Sheet_3.docx]

**S3 File. GSOEP DRM results**

*Continuous income (linear, squared, log)*

When analyzing the GSOEP income measure in its original continuous form as in Hudson and colleagues (2016), again, a null relationship between income and DRM happiness was observed without controls (b=-2.4e-07, 95% CI = -2.5e-06, 2.0e-06) and with controls (b=-8.8e-7, 95% CI = -3.4e-06, 1.7e-06).

The square of income was also not significantly associated with happiness without (b=4.0e-12, 95% CI = -7.4e-12, 1.6e-116) and with controls (b=2.6e-12, 95% CI = 9.3e-12, 1.5e-11), nor was the log of income, again, both without (b=-0.04, 95% CI = -0.12, 0.05) and with controls (b=-0.08, 95% CI = -0.18, 0.01).

*Categorical income*

Results of GSOEP DRM OLS regressions explaining variance in average happiness according to the 16 categories if income are shown in Fig 3 (main text) and Table 1 S3.

**Table 1 S3. Results of OLS regressions in GSOEP DRM data explaining variance in average happiness at the individual level from Income 4 (16 quantiles of income) without and with controls**

|  | | **Happy (without controls)** | | | **Happy (with controls)** | | |
| --- | --- | --- | --- | --- | --- | --- | --- |
| *Income 4* | b | 95% CI | | b | 95% CI | |  |
| Quantile 1 | -0.04 | -0.30 | 0.23 | 0.08 | -0.20 | 0.36 |  |
| Quantile 2 | -0.1 | -0.36 | 0.15 | 0.03 | -0.23 | 0.30 |  |
| Quantile 3 | 0.27 | 0.04 | 0.51 | 0.36 | 0.12 | 0.60 |  |
| Quantile 4 | 0.09 | -0.18 | 0.36 | 0.17 | -0.11 | 0.44 |  |
| Quantile 5 | 0.15 | -0.10 | 0.39 | 0.21 | -0.04 | 0.46 |  |
| Quantile 6 | 0.25 | 0.01 | 0.49 | 0.30 | 0.06 | 0.54 |  |
| Quantile 7 | -0.03 | -0.28 | 0.23 | 0.02 | -0.24 | 0.27 |  |
| Quantile 8 | 0.00 | -0.23 | 0.22 | 0.02 | -0.20 | 0.25 |  |
| Quantile 9 | -0.25 | -0.52 | 0.01 | -0.20 | -0.46 | 0.06 |  |
| Quantile 10 | -0.01 | -0.23 | 0.22 | 0.01 | -0.20 | 0.23 |  |
| Quantile 11 | 0.14 | -0.13 | 0.41 | 0.15 | -0.12 | 0.42 |  |
| Quantile 12 | -0.1 | -0.35 | 0.15 | -0.08 | -0.32 | 0.17 |  |
| Quantile 13 | -0.13 | -0.37 | 0.11 | -0.10 | -0.33 | 0.14 |  |
| Quantile 14 | 0.11 | -0.14 | 0.36 | 0.09 | -0.16 | 0.33 |  |
| Quantile 15 | 0.02 | -0.21 | 0.26 | 0.06 | -0.17 | 0.29 |  |
| Quantile 16 | *reference* | | | *reference* | | |  |
| Constant | 2.9 | 2.7 | 3.1 | 2.6 | 2.2 | 2.9 |  |
| r2 | 0.009 | | | 0.03 | | |  |
| N | 6766 | | | 6766 | | |  |

*Note.* See Table 2 for quantile income values.

In GSOEP DRMs, without controls, those with incomes in the third quantile of €14,472-18K appeared to be happier than those in quantiles 16 (b=0.27, 95% CI = 0.04, 0.51), 15 (b=0.25, 95% CI = 0.04, 0.46), 13 (b=0.40, 95% CI = 0.19, 0.61), 12 (b=0.37, 95% CI = 0.16, 0.58), ten (b=0.28, 95% CI = 0.09, 0.46), nine (b=0.53, 95% CI = 0.30, 0.75), eight (b=0.28, 95% CI = 0.09, 0.46), seven (b=0.30, 95% CI = 0.08, 0.51), two (b=0.38, 95% CI = 0.17, 0.58), and one (b=0.31, 95% CI = 0.08, 0.54Similar results were observed with controls.

*Lowess*

As shown in Fig 4 (main text), there is no evidence of greater happiness associated with greater income after the third quantile (€14,472-18K). It does appear that after quantile 13 (€49,032-54K) there is an increase in happiness, though this increase does not exceed the level of happiness associated with the income category €14,472-18K.

*Splines*

The lowess and spline regressions suggested null results overall, as the variation was small in magnitude.

We fit a spline regression with knots at quartiles corresponding to €19,200, €30K and €42K. Without controls, there was a small positive relationship between income and happiness prior to €19,200 (b=0.00003, 95% CI = 0.000009, 0.00005), a small negative relationship between €19,200 and €30K (b=-0.00002, 95% CI = 0.000009, 0.00004), and smaller, more uncertain coefficients at higher levels of income. Similar results were observed with controls.

When using five quantiles with knots at €18K, €25,200, €34,800 and €48K, there was a small positive relationship between income and happiness without controls prior to €18K (b=0.00003, 95% CI = 0.00005, 0.000007) and similar, more uncertain relationships at higher levels of income and with controls.

We also fit a spline regression with a pre-specified knots at €16,800K (the median value in the range of €14,472-18K in the data) and €52,800 (the median value of the range €49,032-54K).

Prior to €16,800K, there was a small positive relationship between income and happiness without controls (b=0.00003, 95% CI = 0.000002, 0.00005). The relationship was similar and in magnitude and a little less certain with controls (b=0.00002, 95% CI = -0.000003, 0.00005). Between €16,800K and €52,800, there was a small negative relationship between income and happiness (b=-0.000007, 95% CI = -0.00001, -0.000002), which was similar with controls (b=0.000008, 95% CI = -0.00001, -0.000004). The positive coefficient after €52,800 was small without (b=0.000004, 95% CI = -0.0.0000004, 0.000008) and with controls 0.000004, 95% CI = -0.0000007, -0.000008).
