# Supplementary material for: Money Does Not Always Buy Happiness, but Are Richer People Less Happy in Their Daily Lives? It Depends on How You Analyze Income
Source: Front Psychol. 2022 May 31;13:883137. doi: 10.3389/fpsyg.2022.883137 (PMC9199446; doi:10.3389/fpsyg.2022.883137)
Supplement: Supplementary file 4 [file Data_Sheet_4.docx]

**S4 File. GSOEP ESM results**

*Continuous income (linear, squared, log)*

There was no substantive relationship between continuous linear income and happiness in OLS regressions both without (b=2.8e-06, 95% CI = -0.00001, 0.000005) and with controls (b=5.5e-06, 95% CI = -0.00001, 0.000003). This was also the case for income squared without (b=-3.4e-11, 95% CI = -1.2e-10, 5.2e-11) and with controls (b=-5.1e-11, 95% CI = -1.4e-10, 3.8e-11 ), as well as for log income without (b=-0.04, 95% CI = -0.28, 0.20) and with controls (b=-0.15, 95% CI = -0.43, 1.5).

*Lowess*

As shown in Fig 5 (main text), there is no evidence of higher income being associated with greater happiness after around €20K, which is not dissimilar to the figure of €16,800K from the DRMs.

*Splines*

The spline regression with knots at quartiles corresponding to €19,200, €30K and €42K were not substantive within any quartile without or with controls (coefficients were all around 0.00002 in magnitude with 95% CIs containing zero). This was also the case for the spline regression with knots at five quantiles corresponding to €18K, €25,200, €34,800 and €48K. Single knots in three separate models at €16,800K, €20K and €52,800 did not show a substantive relationship between income and happiness before or after the knots both without and with controls.
